# Augmentation index is not a proxy for wave reflection magnitude: mechanistic analysis using a computational model

**DOI:** 10.1152/japplphysiol.00769.2018

**Published:** 2019-05-30

**Authors:** Maarten H. G. Heusinkveld, Tammo Delhaas, Joost Lumens, Wouter Huberts, Bart Spronck, Alun D. Hughes, Koen D. Reesink

**Affiliations:** ^1^CARIM School for Cardiovascular Diseases, Maastricht University, The Netherlands; ^2^Department of Biomedical Engineering, Yale University, New Haven, Connecticut; ^3^Institute of Cardiovascular Science, University College London, United Kingdom

**Keywords:** augmentation index, computational modeling, hemodynamics, wave reflection

## Abstract

The augmentation index (AIx) is deemed to capture the deleterious effect on left ventricular (LV) work of increased wave reflection associated with stiffer arteries. However, its validity as a proxy for wave reflection magnitude has been questioned. We hypothesized that, in addition to increased wave reflection due to increased pulse wave velocity, LV myocardial shortening velocity influences AIx. Using a computational model of the circulation, we investigated the isolated and combined influences of myocardial shortening velocity *v*_s,LV_ and arterial stiffness on AIx. Aortic blood pressure waveforms were characterized using AIx and the reflected wave pressure amplitude (${\hat{p}}_{\text{bw}}$, obtained using wave separation analysis). Our reference simulation (normal *v*_s,LV_ and arterial stiffness) was characterized by an AIx of 21%. A realistic reduction in *v*_s,LV_ caused AIx to increase from 21 to 42%. An arterial stiffness increase, characterized by a relevant 1.0 m/s increase in carotid-femoral pulse wave velocity, caused AIx to increase from 21 to 41%. Combining the reduced *v*_s,LV_ and increased arterial stiffness resulted in an AIx of 54%. In a multistep parametric analysis, both *v*_s,LV_ and arterial stiffness were about equal determinants of AIx, whereas ${\hat{p}}_{\text{bw}}$ was only determined by arterial stiffness. Furthermore, the relation between increased AIx and LV stroke work was only ≈50% explained by an increase in arterial stiffness, the other factor being *v*_s,LV_. The ${\hat{p}}_{\text{bw}}$, on the other hand, related less ambiguously to LV stroke work. We conclude that the AIx reflects both cardiac and vascular properties and should not be considered an exclusively vascular parameter.

**NEW & NOTEWORTHY** We used a state-of-the-art computational model to mechanistically investigate the validity of the augmentation index (AIx) as a proxy for (changes in) wave reflection. In contrary to current belief, we found that LV contraction velocity influences AIx as much as increased arterial stiffness, and increased AIx does not necessarily relate to an increase in LV stroke work. Wave reflection magnitude derived from considering pressure, as well as flow, does qualify as a determinant of LV stroke work.

## INTRODUCTION

**Fig. 1.**
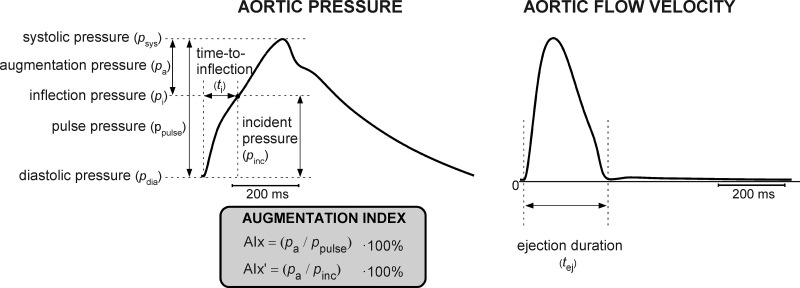
Overview of characteristics and indices extracted from the simulated aortic pressure waveform (*left*) and aortic flow waveform (*right*). *Left*: augmentation index (AIx), calculated as a percentage-fraction using augmentation pressure (*p*_a_) and pulse pressure (*p*_pulse_). AIx′ was calculated as the percentage ratio using augmentation pressure (*p*_a_) and incident pressure (*p*_inc_). Time-to-inflection (*t*_i_) is defined as the time interval between foot and the inflection point at inflection pressure *p*_i_. *Right*: left ventricular ejection duration (*t*_ej_), the time interval during which blood is ejected into the aorta.

The augmentation index (AIx) is defined as the late systolic boost in the aortic pressure waveform divided by pulse pressure and is often expressed as a percentage (26) (Fig. 1). Pressure waveform augmentation is commonly assumed to result from the superposition of a reflected wave originating from a (discrete) reflection site in the periphery onto the incoming pressure wave generated by the heart (26). With increased arterial stiffness, the reflected wave will propagate with increased velocity, causing the augmentation to occur earlier in systole with a consequent increase in AIx (26). AIx is considered a “vascular” parameter intended to quantify the deleterious effect of systolic wave reflection on cardiac workload, which is associated with adverse cardiovascular outcomes (12). An advantage of AIx is its nondimensionality, requiring neither calibration of blood pressure nor measurement of blood flow velocity (19). Blood pressure waveforms can be obtained using noninvasive tonometry at the location of the carotid or radial arteries, or by oscillometric blood pressure recordings at the brachial level (18, 20). Subsequently, AIx can be estimated from a synthesized central pressure waveform derived from a reconstruction algorithm, applied to the tonometric or oscillometric waveforms. The accuracy of noninvasive AIx estimation by tonometry and oscillometry was evaluated by Chen et al. (8) and Horvath et al. (18), finding good correlation between catheter and noninvasive AIx. Furthermore, Wilkinson et al. (44) and Savage et al. (33) reported good interobserver reproducibility of AIx, as measured by tonometry.

The comprehensive meta-analysis of Baksi et al. (5) described AIx to drastically increase with age, despite only a small advance in time of arrival of the reflected wave. Considering the often used paradigm in clinical studies that AIx is a proxy for wave reflection magnitude (12, 19), one would expect similar proportions in the correlation between age and AIx and wave arrival time.

Mechanistically, during systole, the blood pressure waveform is the result of the instantaneous interaction between heart and arterial system. This is illustrated by the fact that aortic and left ventricular pressure patterns are in phase and closely related during LV ejection, when assuming normal aortic valve function (9, 42). Moreover, acute changes in arterial load are directly reflected in the time course of ventricular and aortic pressures (21). However, the potential influence of cardiac contractile properties on the AIx is rarely considered in clinical-epidemiological studies. Though a few clinical studies reported an “association” between systolic augmentation and LV diastolic dysfunction (40), the direct “linkage” between AIx and cardiac contractile function is difficult to determine, since AIx is likely to be confounded by other factors such as heart rate, body height, and mean arterial pressure (12, 34, 36). To overcome this issue, physics and physiology-based models of the cardiovascular system may be employed to evaluate the effect of isolated changes of cardiac and vascular properties on AIx.

The purpose of the present study is to assess the effect of changes in LV contractility and arterial stiffness on AIx, utilizing the CircAdapt computational model of the circulation (www.circadapt.org) (22, 39). This closed-loop model was chosen because of its realistic cardiac mechanics model, with sarcomere length coupled to myofiber stress. Furthermore, it contains a vascular model, simulating arterial and venous pressure and flow wave hemodynamics. First, we modulated LV contractile properties by varying sarcomere shortening velocity (*v*_s_*_,_*_LV_) in the model, and then, we modulated arterial stiffness by varying the vessel stiffness parameter (*k*). Both *v*_s_*_,_*_LV_ and *k* are physiologically relevant parameters, since decreases in *v*_s_*_,_*_LV_ and increases in *k* have been associated with aging (1, 14, 16). We compared AIx to alternative indices of wave reflection derived using established wave separation (WS) analysis (30, 31). Finally, we evaluated the relation between AIx and cardiac workload with respect to variations in LV shortening velocity and arterial stiffness.

## METHODS

### 

#### The CircAdapt model.

We used the CircAdapt computational model, a closed-loop model of the four-chambered heart and circulation, to simulate cardiac mechanics and hemodynamics (22, 39).

**Fig. 2.**
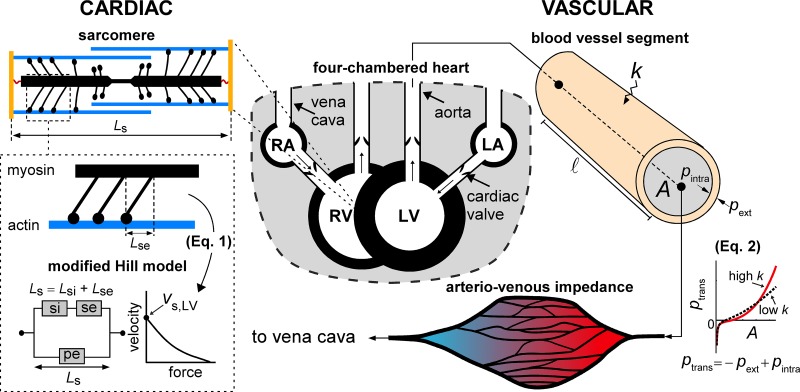
Overview of the primary relevant modules of the CircAdapt model. We distinguish two modules; the module underlying mechanics of myocardial tissue (CARDIAC) and the module underlying wave propagation in the vascular tree (VASCULAR). The sarcomere model consists of a contractile (si) element with length *L*_si_ and a series-elastic (se) element with length *L*_se_, in parallel with parallel-elastic element (pe) with sarcomere length *L*_s_, according to the model of Hill, which assumes a force-velocity relation as shown. Blood vessels are characterized by a stiffness coefficient (*k*), and intraluminal-, external- and transmural pressure (*p*_intra_, *p*_ext_, and *p*_trans_, respectively), and cross-sectional area (*A*). A vessel segment with a given length (*l*) connects a proximal node to a distal node. Peripheral vascular beds are modeled using an arteriovenous impedance model. LA, left atrium; LV, left ventricle; RA, right atrium; RV, right ventricle. The model simulation code can be downloaded at https://zenodo.org/record/3066179.

CircAdapt consists of a limited number of modules representing cardiac walls, valves, arteries, and veins, as well as the systemic and pulmonary vascular beds. The modules directly relevant for the present study are highlighted in Fig. 2.

Briefly, CircAdapt’s contraction model constitutes a modified Hill model, describing the sarcomere mechanics (Fig. 2). The sarcomere model contains a contractile element with length *L*_si_, arranged in series to a series-elastic element with length *L*_se_, which, in turn, is arranged parallel to a passive-elastic element with length *L*_s_ (Fig. 2). The contractile and series-elastic elements describe the additional stress generation due to muscle activation, whereas the passive-elastic element describes passive muscle behavior (Fig. 2). Moreover, the length of the series-elastic element can be interpreted as the elastic deformation of the myosin heads in response to load (22). Total sarcomere length is defined as the sum of the length of the contractile element and series element, i.e., *L*_s_ = *L*_si_ + *L*_se_. The rate of shortening of the contractile element (d*L*_si_/d*t*) is given by

(1) $$\frac{\text{d}L_{\text{si}}}{\text{d}t}=v_{\text{s},\text{LV}}\left[\left(L_{\text{se}}-L_{\text{se},\text{iso}}\right)/L_{\text{se},\text{iso}}\right],$$

with *v*_s_*_,_*_LV_ LV sarcomere shortening velocity, assumed 7 µm/s (13) and *L*_se,iso_ the length of the series elastic element at the onset of isovolumetric contraction. In the model, *v*_s_*_,_*_LV_ scales d*L*_si_/d*t*. A phenomenological mechanical activation parameter *C* is calculated as a function of *L*_s_, *L*_si_, and time (22, 39). Actively generated myofiber stress is determined using the mechanical activation, multiplied by the sarcomere extension from reference [i.e., *C* (*L*_si_ *− L*_si_*_,_*_ref_)] (39). To obtain cavity pressures and volumes, CircAdapt uses the one-fiber model of Arts et al. (3) that allows for separation of the contribution of cardiac geometry and myofiber stress to cardiac cavity pressure, assuming myofiber stress to be homogeneously distributed within the myocardial wall. Furthermore, the heart is enclosed by the pericardium, modeled as a compliant sac (28) (Fig. 2).

CircAdapt enables simulation of pressure and flow waves in arteries and veins. We implemented an arterial and venous tree describing the aorta and vena cava, as well as the subclavian-, carotid-, brachial-, and femoral arteries and veins, respectively. Transmural pressure (*p*_trans_) and cross-sectional lumen area (*A*) are related nonlinearly, expressed by the constitutive law based on Arts et al. (4)

(2) $$p_{\text{trans}}\left(A\right)=-p_{\text{ext}}+p_{0}\left(\left(1+b\right){\left(\frac{A}{A_{0}}\right)}^{1+\frac{k/3-2}{1+b}}-\frac{bA_{0}}{A}\right),$$

with *p*_0_ denoting reference pressure, *A*_0_ denoting reference cross-sectional area, and *k* representing the vessel stiffness coefficient, governing the nonlinearity of the pressure-area relation, and, thereby, material stiffness (Fig. 2) (4). Additionally, parameter *b* was incorporated to avoid collapse of the tube with negative transmural pressure. Furthermore, *p*_ext_ represents a prescribed external pressure. For our reference simulation, we chose *k* values of 8 for the aorta and vena cava. For both of the arteries and veins, we chose *k* values of 14 for the subclavian, 16 for the carotid, 18 for the brachial, and 20 for the femoral, describing elastic taper [i.e., the increase in vessel wall stiffness toward the periphery (43)]. Furthermore, estimations for *A*_0_ and vessel length were based on human data given in Westerhof et al. (43). Peripheral vascular beds were modeled using an arteriovenous impedance model (2).

#### Simulation protocols.

Departing from our reference (REF) situation as detailed above, we simulated three additional scenarios:

- Reduced shortening velocity (*v*_s_*_,_*_LV_) of LV sarcomeres from 7 µm/s to 3 µm/s (“reduced shortening velocity”; *v*_s_*_,_*_LV_*↓*, Table 1). To characterize the imposed change in *v*_s_*_,_*_LV_, we calculated peak systolic strain rate, because peak systolic strain rate is considered a strong measure of LV contractility (15).
- Increased stiffness of arteries by increasing stiffness parameter *k* (“arterial stiffening”; *k↑*, Table 1). Because the modeled arterial tree contains segments with different *k* values, *k* values of all segments were increased by addition of a factor ∆*k*. Here, ∆*k* was set to equal 12 (i.e., *k*_stiffening_ = *k*_REF_ + 12). According to clinical standards, we quantified arterial stiffness using pulse wave velocity. We obtained pulse wave velocity from transit time and travel distance. Transit time was calculated by identification of the foot of the carotid and femoral pressure waveforms. To identify the foot of the pressure wave, we used the maximum of the second-order derivative (10). Travel distance, on the other hand, was calculated as the path difference between the combined length of the aortic to femoral artery segments and aortic to carotid artery segments.
- The combined effect of a change in cardiac and vascular tissue properties (*combined*; *k↑*, *v*_s_*_,_*_LV_*↓*, Table 1).

**Table 1.** Influence of LV shortening velocity and arterial stiffness on blood pressure indices and reflection indices for four distinct simulated scenarios

Simulation→ Metric↓	Reference (REF)	Reduced Shortening Velocity (*v*_s,LV_)↓	Arterial Stiffening (*k*↑)	Combined (*v*_s,LV_↓, *k*↑)	Unit
*p*_sys_	127	126	162	158	mmHg
*p*_dia_	60	63	42	42	mmHg
*p*_pulse_	66	63	120	116	mmHg
*p*_mean_	92	92	92	92	mmHg
AIx	21	42	41	54	%
AIx′	27	72	70	120	%
${\hat{p}}_{\text{bw}}$*/p^fw*	76	74	76	76	%
p^bw	28	26	51	48	mmHg
Time-to-inflection	96	83	69	60	ms
Ejection duration	238	274	285	309	ms
LV stroke work	1.04	0.98	1.18	1.1	J
PSSR	–0.91	–0.69	–0.80	–0.64	s^−1^

The indices *p*_sys_, *p*_dia_, *p*_pulse_, and *p*_mean_ are systolic, diastolic, systolic-diastolic, and mean aortic blood pressure, respectively, and PSSR denotes peak systolic strain rate. Note that AIx′ and ${\hat{p}}_{\text{bw}}$*/${\hat{p}}_{\text{fw}}$* are wave reflection indices that consider the ratio of backward and forward pressure wave amplitudes, while AIx considers the fraction of augmentation pressure and pulse pressure.

Additionally, to evaluate the dependence of AIx on LV shortening velocity and arterial stiffness, we also performed parameter sweeps of *v*_s_*_,_*_LV_ and ∆*k*, varying LV shortening velocity *v*_s_*_,_*_LV_ from 2 to 10 µm/s with 1 µm/s increments and ∆*k* from *−*2 to 14 with increments of 1. All simulations were performed in MATLAB 2015a (The Mathworks, Natick, MA). In all simulations, mean arterial pressure and cardiac output were maintained at 92 mmHg and 5.1 l/min, respectively, through changes of systemic vascular resistance and circulating blood volume, while heart rate was kept constant at 72 beats/min. Collapsible tube fraction *b* was kept at 0.02. Hemodynamics reached steady state before analyses. The underlying simulation code and analysis programs are available under the GNU General Public License v3.0 and can be downloaded from GitHub (https://github.com/Mheu1991/AugmentationIndex_SimulationStudy), and at https://zenodo.org/record/3066179.

#### Wave reflection indices extracted from simulations.

Wave reflection indices were computed for simulated pressure and flow velocity waveforms of the aorta. To benchmark the relationships between simulated cardiovascular alterations and AIx, we included wave reflection indices obtained using wave separation (WS) analysis ((30), detailed below). While WS analysis yields a wave reflection index expressed as a percentage ratio, AIx expresses wave reflection as a percentage fraction using the expression in Fig. 1 (12). Hence, we also introduce the parameter AIx′, defined as a percentage ratio of augmentation pressure to incident pressure (i.e., *p*_a_/*p*_inc_ · 100%). Furthermore, we calculated time-to-inflection (*t*_i_, Fig. 1, *right*), defined as the time interval between the foot of the pressure waveform and inflection point (19). Locations of foot and inflection were identified using the peak and zero-crossing of the second-order derivative of the pressure curve. We extracted LV ejection duration to evaluate whether it was influenced by our simulated changes in LV shortening velocity and arterial stiffness. Using the second-order derivative of the aortic valve flow velocity, LV ejection duration was calculated as the time difference between the beginning and end of ejection (Fig. 1, *right*, vertical dashed lines).

The concept of WS analysis is based on solving the one-dimensional equations of mass conservation and momentum balance in elastic tubes (30). In WS analysis, instantaneous changes in pressure and flow velocity, representing wave fronts, are calculated for the forward and backward directions using the water-hammer equations:

$$\text{d}p_{\text{fw}}=\text{ρ}c\text{d}U_{\text{fw}},$$

and

(3) $$\text{d}p_{\text{bw}}=-\text{ρ}c\text{d}U_{\text{bw}},$$

where d*p*_fw_ and d*U*_bw_ denote forward or backward wave fronts of pressure and flow velocity, respectively, whereas ρ is the blood density (1050 kg/m^3^), and *c* is the pulse wave velocity. The variables d*p*_fw_, d*p*_bw_, d*U*_fw_, d*U*_bw_, and *c* were determined, as described by Parker (30). Through calculation of forward and backward pressures *p*_fw_ and *p*_bw_, obtained by integration of separated instantaneous pressure components, we derived wave reflection index ${\hat{p}}_{\text{bw}}$/${\hat{p}}_{\text{fw}}$, defined as the percentage ratio between backward pressure wave amplitude (${\hat{p}}_{\text{bw}}$) and forward pressure wave amplitude (${\hat{p}}_{\text{fw}}$).

#### Calculation of cardiac workload.

To assess whether there is a relation between increased AIx and increased cardiac external work, we assessed cardiac workload by calculation of LV stroke work (*W*_stroke_). The variable *W*_stroke_ was calculated as the numerically integrated area contained by the LV pressure (*p*_LV_)–volume (*V*_LV_) relationship:

(4) $$W_{\text{stroke}}=\oint p_{\text{LV}}\text{d}V_{\text{LV}}.$$

#### Statistical analysis.

Statistical analyses were performed using IBM SPSS Statistics for Windows, version 24 (IBM Corp., Armonk, NY). Linear regression analysis was performed to examine the relationship between wave characteristics. *P* < 0.05 was considered statistically significant.

## RESULTS

### 

#### Effects of reduced LV shortening velocity and increased arterial stiffness on augmentation index.

**Fig. 3.**
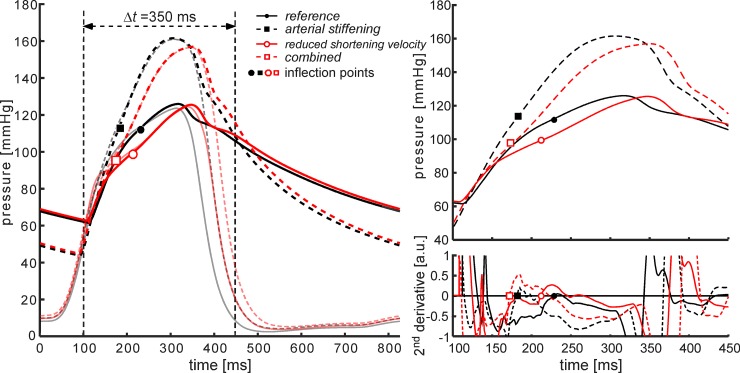
Aortic and left ventricular pressure curves for the four simulated scenarios. *Left*: overview of simulated left ventricular and aortic pressure waveforms for four simulated scenarios. *Right*: closer look at the systolic segment (*t* = 100–450 ms) of the pressure waveforms (*top*) and their second-order derivatives (*bottom*). *Right*: inflection points (black and red symbols, respectively) are located by determining the zero-crossing of the second-order derivative of each simulated waveform.

Figure 3 and Table 1 contain an overview of pressure waveforms and derived characteristics for the four simulated scenarios. Simulating *v*_s_*_,_*_LV_*↓* (red solid curve) did not affect absolute pressure values (Table 1). However, the inflection in the pressure waveform came earlier (from 96 to 83 ms, Table 1), after the foot of the pressure waveform with a marked increase in AIx (from 21 to 42%, Table 1). LV ejection duration was prolonged, with respect to the reference situation (from 238 to 274 ms, Table 1), and peak systolic strain rate (PSSR) magnitude decreased (from –0.91 s^–1^ to –0.69 s^–1^; Table 1). Simulating *k↑* (Fig. 3, dashed black line), increased systolic pressure (127 to 162 mmHg) and decreased diastolic pressure (60 to 42 mmHg; Table 1). The inflection occurred significantly earlier during systole (time-to-inflection decreased from 96 to 69 ms), and AIx was markedly increased (21 to 41%, Table 1). The PSSR magnitude was only moderately decreased from –0.91 s^–1^ to –0.80 s^–1^ (Table 1). The combined effect of *k↑* and *v*_s_*_,_*_LV_*↓* resulted in a waveform that can be considered an intermediate (Fig. 3, red dashed curve) curve of the *k↑* and *v*_s_*_,_*_LV_*↓* curves, with an AIx increase from 21 to 54%. Ejection duration increased to 309 ms, and the inflection point occurred 60 ms after the foot of the pressure waveform. Simulating *k↑* and *v*_s_*_,_*_LV_*↓* invoked the largest reduction in PSSR magnitude, i.e., reducing from –0.91 s^–1^ to –0.64 s^–1^ (Table 1).

#### Effects on alternative measures of wave reflection.

**Fig. 4.**
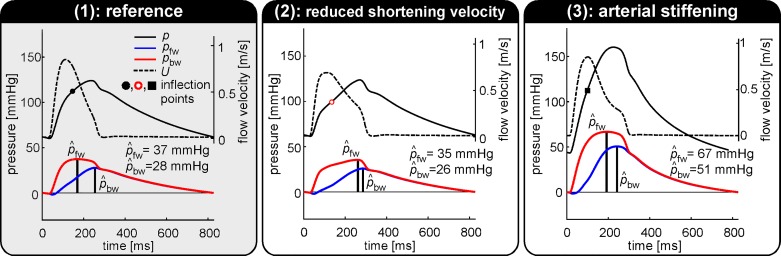
Wave separation analysis performed using pressure (*p*) and flow velocity (*U*) waveforms obtained from *1*) the reference simulation, *2*) the reduced shortening velocity simulation, and *3*) the arterial stiffening simulation. Wave reflection index ${\hat{p}}_{\text{bw}}$*/${\hat{p}}_{\text{fw}}$* was calculated as the percentage ratio of backward pressure wave amplitude (${\hat{p}}_{\text{bw}}$) and forward pressure wave amplitude (${\hat{p}}_{\text{fw}}$) using wave separation analysis. The simulated aortic blood pressure waveform can be reconstructed by the addition of separated pressure components (*p*_fw_ and *p*_bw_). The offset is determined by the pressure at *t* = 0.

**Fig. 5.**
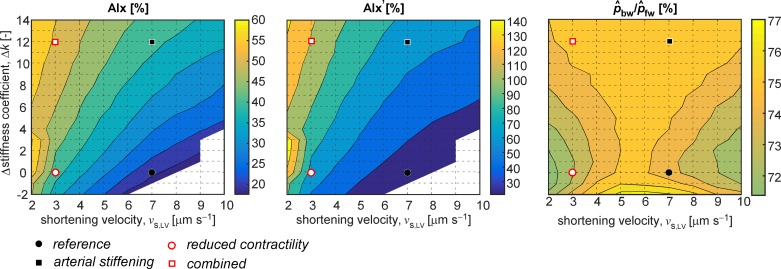
Contour plots indicating the relationship between the changes in LV shortening velocity and arterial stiffness and the wave reflection indices considered: AIx (*left*), AIx′ (*middle*), and ${\hat{p}}_{\text{bw}}$*/${\hat{p}}_{\text{fw}}$* (*right*). The distinct simulated scenarios are highlighted by the circles and squares, as indicated. Each grid point corresponds to a single simulation. LV shortening velocity parameter *v*_s_*_,_*_LV_ was varied from 2 µm/s to 10 µm/s with 1 µm/s increments, and arterial stiffness parameter *∆k* was varied between –2 and +14 with increments of 1.

Figure 4 contains a breakdown of simulated pressure waveforms into forward (*p*_fw_) and backward (*p*_bw_) pressure wave components, using wave separation (WS) analysis. Furthermore, separated pressure wave amplitudes, ${\hat{p}}_{\text{fw}}$ and ${\hat{p}}_{\text{bw}}$ are indicated for the REF (Fig. 4, *left*), *v*_s_*_,_*_LV_*↓* (Fig. 4, *middle*), and *k↑* (Fig. 4, *right*) simulations. Reducing LV shortening (*v*_s_*_,_*_LV_) caused *p*_fw_ to change in shape but not in amplitude as compared with the reference situation. On the contrary, no clear difference was observed in the *p*_bw_ waveform. As a result, reflection index ${\hat{p}}_{\text{bw}}$/${\hat{p}}_{\text{fw}}$ virtually remained unchanged (76 and 74%, respectively, Table 1). Increased arterial stiffness (*k↑*) resulted in an increase in backward pressure wave amplitude (${\hat{p}}_{\text{bw}}$) from 28 to 51 mmHg, whereas ${\hat{p}}_{\text{bw}}$/${\hat{p}}_{\text{fw}}$ remained unchanged (76 for both REF and *k↑*, Table 1). The latter is attributable to a proportional increase in ${\hat{p}}_{\text{fw}}$ and ${\hat{p}}_{\text{bw}}$ (Fig. 5).

#### Parameter sweeps.

Parameter sweeps of *v*_s_*_,_*_LV_ and ∆*k* provide more detailed insight into the relationships between the changes in cardiac and vascular properties and the indices AIx and ${\hat{p}}_{\text{bw}}$/${\hat{p}}_{\text{fw}}$. Combined changes in cardiac and vascular tissue properties seem to relate nonlinearly to observed changes in AIx, as is apparent in the contour plot of AIx as a function of *v*_s_*_,_*_LV_ and ∆*k* (Fig. 5, *left*). Our simulation results indicate that AIx is monotonically increasing, with both higher arterial stiffness values and lower LV sarcomere shortening velocities. For the simulations corresponding to the white regions (i.e., high *v*_s_*_,_*_LV_ and low *k* values), no inflection point was present in the systolic phase of the aortic blood pressure curve. The relation between AIx′ and changes in *v*_s_*_,_*_LV_ and *k* was not different, albeit that absolute values, expressing pressure wave augmentation as the ratio between pressure above the inflection point on the curve to pressure below the inflection point, ranged from 30 to 140% for all simulations (Fig. 5, *middle*). Using wave separation, we did not find distinctive differences in the ratio between backward and forward pressure amplitude ${\hat{p}}_{\text{bw}}$/${\hat{p}}_{\text{fw}}$ (range 72 to 76%, Fig. 5, *right*). This finding was caused by almost proportional increases in ${\hat{p}}_{\text{fw}}$ and ${\hat{p}}_{\text{bw}}$.

#### Relationship between AIx and left ventricular stroke work.

**Fig. 6.**
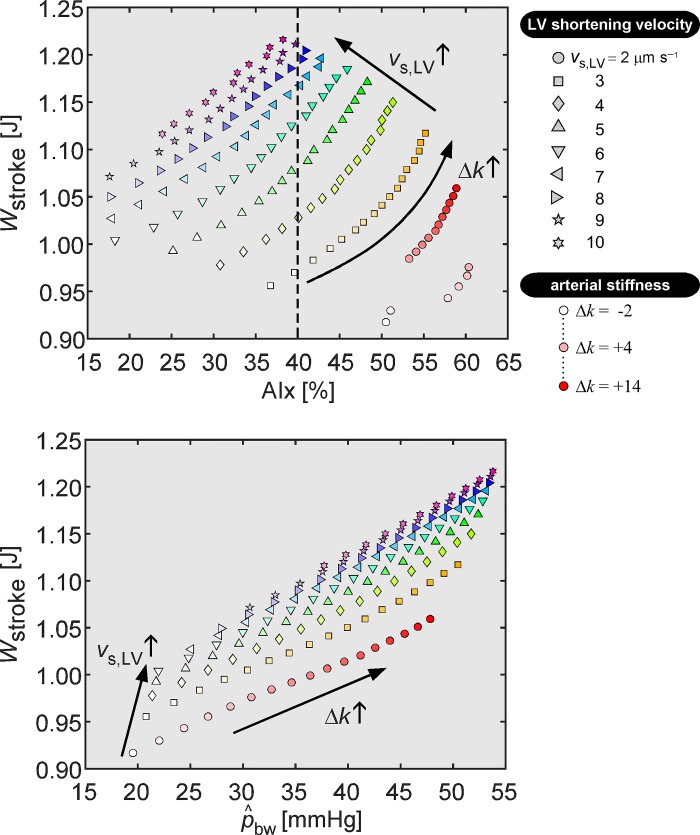
Relation between left ventricular stroke work (*W*_stroke_) and wave reflection indices, i.e., augmentation index (AIx) and backward pressure amplitude (${\hat{p}}_{\text{bw}}$), respectively. Each symbol represents a simulation with a particular left ventricular sarcomere shortening velocity (*v*_s_*_,_*_LV_; indicated by symbol type) and imposed arterial stiffness (*∆k*; indicated using symbol color saturation). *Top*: scatterplot indicating the relation between AIx and *W*_stroke_. The dashed line illustrates variation in *W*_stroke_ values at an AIx value often found in elderly patients (see text). *Bottom*: scatterplot displaying the relation between ${\hat{p}}_{\text{bw}}$ as determined using wave separation and *W*_stroke_. Arrows indicate the direction of changes in *v*_s_*_,_*_LV_ and *∆k* for the simulations.

Figure 6 (*top*) shows the relation between AIx and *W*_stroke_ from simulations with varying arterial stiffness (indicated by color saturation coding) and LV shortening (indicated by symbol type coding). Parameter sweeps revealed a large scatter in the relation between AIx and *W*_stroke_ (Fig. 6). At a fixed AIx of 40%, typically found in older subjects (25), *W*_stroke_ varies by as much as 25% (Fig. 6, dashed line). On the other hand, scatter in the relation between ${\hat{p}}_{\text{bw}}$ and *W*_stroke_ was smaller as compared with that in the relation between AIx and *W*_stroke_ (*≤*11% variation in *W*_stroke_, for a given value of ${\hat{p}}_{\text{bw}}$), suggesting an increased cardiac workload with increasing backward pressure wave amplitude (Fig. 6).

## DISCUSSION

To our knowledge, this is the first study to mechanistically study and discriminate the influence of LV contractile and arterial stiffness properties on the augmentation index. We compared our findings with alternative measures of wave reflection that were derived using wave separation analysis.

### 

#### Key findings and interpretation.

We found that the AIx is dependent on vascular, as well as cardiac properties (Fig. 5). Moreover, increased AIx did not necessarily relate to increased LV workload (Fig. 6), which goes against the commonly used concept of increased LV workload by increased LV afterload as caused by earlier systolic wave reflections.

We explain the observed cardiac impact on AIx as follows. Given a constant stroke volume, a decreased myocardial shortening velocity will decrease LV volumetric rate (LV d*V*/d*t*) in early systole, causing a greater portion of stroke volume to be expelled during late systole. Therefore, the pressure increase before the inflection point will be relatively smaller than the pressure increase after the inflection point, which leads to an increased AIx. This assumes that the timing of the inflection point is primarily determined by the complex arterial impedance, which is in line with current thinking (5).

The AIx is calculated from an aortic blood pressure waveform, whereas wave separation analysis uses, in addition to blood pressure, flow (velocity) information and takes into account aortic impedance (30). This may explain why backward pressure amplitude in our study is a better correlate of wave reflection, while AIx characterizes rather the interactive influence of heart and blood vessels on the blood pressure.

Hemodynamic interaction between blood vessels and heart has been reported in patients, as judged from changes in peak systolic strain rate in response to an imposed afterload change (7). Additionally, previous experimental papers suggest that acute changes in LV myocyte external load (i.e., arterial impedance) impacts myocardial shortening (21, 32), corroborating that cardiac properties by themselves and/or in response to vascular changes influence AIx.

Taken together, the AIx may be an integrative marker of concurrent degenerative processes leading to increased arterial stiffness and reduced myocardial shortening velocity. Clearly, such reinterpretation of AIx requires further corroboration from clinical studies using independent cardiac and vascular measurements. As discussed in the meta-analysis of van der Waaij et al. (38), the number of clinical studies reporting effects of chronic changes in arterial stiffness on LV function is limited.

#### Choice of model parameters.

Clearly, AIx will likely be determined by multiple factors, especially in humans. For our modeling study, we intentionally selected only one cardiac (*v*_s_*_,_*_LV_) and one vascular (*k*) parameter with demonstrated linkages to tissue properties (myocardial shortening velocity and arterial stiffness exponent). The simulated changes in *v*_s_*_,_*_LV_ and *k* were by no means intended to model the clinical-epidemiological profiles of heart failure or vascular aging. The imposed reduction in LV shortening velocity (*v*_s_*_,_*_LV_*↓*) significantly changed the peak systolic strain rate from *−*0.91 s^–1^ for the reference simulation to *−*0.69 s^–1^ (i.e., a reduction in magnitude of 24%). This decrease in peak systolic strain rate is physiologically plausible, as based on reference data reported in Dalen et al. (11). In previous work, we conducted a local sensitivity analysis assessing how changes in cardiac CircAdapt model parameters affect aortic augmentation index (17). In this analysis, testing four candidate cardiac parameters, we found *v*_s_*_,_*_LV_ to be the most important cardiac determinant of augmentation index, which motivated us to select this model parameter in our analysis. We chose to reduce *v*_s_*_,_*_LV_ based on rat experimental data reporting force-velocity relations in papillary muscle (1). These data showed a linear decrease in peak muscle shortening velocity of *≈*50% with increasing age (i.e., 100 to 1,000 days). Moreover, in isolated human ventricular myocytes, a reduction in shortening velocity with age was also observed (16). The arterial stiffening simulation (*k↑*) corresponded to a relevant carotid-to-femoral pulse wave velocity increase from 6.6 m/s to 7.6 m/s.

#### Alternative metrics in relation to wave reflection magnitude.

**Fig. 7.**
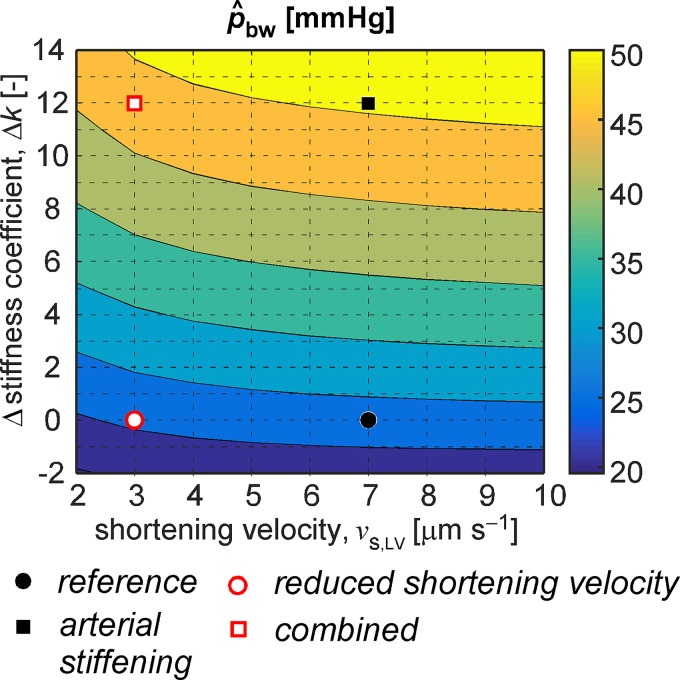
Effect of changes in sarcomere shortening velocity *v*_s_*_,_*_LV_ and vessel stiffness (*k*) on backward pressure amplitude (${\hat{p}}_{\text{bw}}$) assessed using wave separation (WS) analysis. The distinct simulated scenarios are highlighted by the dots and squares as indicated. Each grid point corresponds to a single simulation.

Augmentation index expressed as a percentage ratio (i.e., the parameter AIx′) showed the same dependency on vascular and cardiac properties, as was found for the regular AIx. In contrast to AIx and AIx′, the ratio of backward and forward pressure wave amplitude calculated by means of wave separation analysis remained relatively unaffected for all simulations (Fig. 5). This was caused by a proportional increase in forward pressure wave amplitude with increased arterial stiffness. Such increased forward pressure wave amplitude with age was also observed in men and women in the Framingham Heart Study and was associated with age-related stiffening of central arteries (37). Our simulations did show a clear pattern between arterial stiffness and backward wave amplitude (${\hat{p}}_{\text{bw}}$), as determined using wave separation analysis. Moreover, the pattern was independent of LV sarcomere shortening velocity (Fig. 7). Potential implications of increased backward wave amplitude with respect to cardiovascular risk were reported by Weber et al. (41). Two key findings of their study were that *1*) backward wave amplitude was the most consistent predictor of a composite cardiovascular end point, including mortality, in a group of 725 patients undergoing coronary angiography, and *2*) the predictive value of AIx and augmentation pressure was inferior to that of backward wave amplitude (41). The present study supports their findings and extends the explanation toward cardiac influences.

One should take caution when interpreting our simulated scenarios as emulators of human aging and, hence, to expect an increased reflection magnitude as has been reported by Segers et al. (35) in the aging population. Several aspects regarding cardiovascular structure and function (e.g., cardiac mass and vascular segment diameters), which reportedly change with age, have been kept constant throughout our simulations. Previously, Maksuti et al. (24) and Pagoulatou and Stergiopulos (27) performed simulation studies focusing on changes in arterial hemodynamics during physiological aging, whereas Willemet et al. (45) introduced a method to generate virtual cohorts consisting of arterial hemodynamics models, with varying population characteristics, including age. Future studies on the evolution of AIx and other wave reflection indices may consider these methods as a take-off point.

Simulations revealed a linear association between ejection duration and augmentation index. On the basis of the simulations obtained in our multistep parametric analysis, an increase in ejection duration of 20 ms caused AIx to increase with nine percentage points (i.e., indicated by an unstandardized *B* of 0.43%/ms, 95% confidence interval = [0.40, 0.45] %/ms, *P* < 0.001). In the study of Sharman et al. (36), a similar association between ejection duration and augmentation index of 10 percentage points per 20 ms of increase in ejection duration was found. In our model, this association is explained by a decrease in LV early systolic volumetric rate with constant stroke volume, causing a significant portion of the LV stroke volume to be expelled after the inflection point (see above).

#### Cardiac workload.

In the present study, we quantified cardiac workload by calculating left ventricular stroke work. Increasing arterial stiffness caused left ventricular stroke work to increase (+13%). Left ventricular stroke work slightly decreased (*−*6%) for the simulation with reduced left ventricular shortening velocity (Table 1). While AIx is derived from arterial measurements, using it to quantify cardiac workload appears to be erroneous, as cardiac contractility plays a role as well. Results imply that backward pressure amplitude—rather than depending solely on arterial stiffness in these simulations—to be more indicative of cardiac workload than AIx.

#### Previous clinical studies on ventriculo-arterial interaction.

Below, two examples of clinical studies investigating aspects of ventriculo-arterial interaction are discussed. Bell et al. (6) reported that during systole, the proximal aorta elongates, imposing a higher stretch-related workload on the LV. During diastole, the amount of elastic recoil energy is positively associated with increased early diastolic LV filling, suggesting that elastic recoil of the proximal aorta may benefit LV diastolic function through mechanical ventriculo-arterial coupling. In their analyses on the relations between aortic and LV measures, statistical correction of the (potential) confounding effect of wave reflections was performed using AIx. Given the present results, adjusting for AIx as a proxy of wave reflection may lead to overcorrection or undercorrection for the cardiac influence.

Palombo et al. (29) investigated the treatment effects of a calcium-channel blocker on LV structure (e.g., LV mass) and function (e.g., LV stroke work) indices, as well as central (aortic) hemodynamics indices. Findings from the study included that the ratio of backward pressure amplitude and forward pressure amplitude was equal between hypertensive patients and normotensive controls, while backward pressure amplitude and LV stroke work were significantly higher for the hypertensive patient group. These findings corroborate our simulation findings that ${\hat{p}}_{\text{bw}}$ best reflects the impact of backward wave reflection with increased arterial stiffness on LV workload.

#### Limitations.

When interpreting our results, one has to realize that the data presented were not based on patient measurements but come from computer simulations. Although we did not fit our model to patient data, the order of magnitude of the AIx compares well with human studies. In our reference simulation, AIx was found to be equal to 21%, which is in accordance with the range of values reported by Hughes et al. (19) in 65 healthy individuals (44 *±* 14 yr) and by Savage et al. (33) in 188 patients (56 *±* 15 yr) with chronic renal failure. Imposing either reduced LV shortening velocity or increased arterial stiffness caused AIx to increase to 41 and 42%, respectively. This is equivalent to the AIx measured in subjects between 70 and 80 years of age (25).

The following aspects regarding model assumptions and choices warrant discussion: The CircAdapt model used in this study describes a highly simplified cardiac geometry, relating myofiber stress and strain by single values. This approach was shown to be valid under the assumption that myofiber stress is homogeneously distributed (3). A previous study using the CircAdapt model obtained physiologically realistic LV strain patterns, as compared with global LV strain patterns obtained from MR tagging (23). Therefore, we believe CircAdapt’s contraction model is appropriate for our study’s purpose.

Furthermore, the wave propagation model we used neglects nonlinear convective acceleration (i.e., we assume linear wave behavior), thereby introducing a small modeling error in pressure and flow waveforms. However, the implication of such an error to derived hemodynamic indices is negligible in our study, since all of the wave reflection indices we derived also assume linear wave behavior.

We simulated arterial stiffening by increasing material stiffness parameter *k* (*Eq. 2*). Mean arterial pressure (i.e., *p*_0_) was assumed to remain constant. As such, systolic blood pressure increased, whereas diastolic blood pressure decreased, which may not be representative for patients for which diastolic blood pressure is increased as well, e.g., due to increased peripheral resistance. To assess the implication of keeping mean arterial pressure constant for our findings, we repeated the reference, reduced shortening velocity, arterial stiffening, and combined simulations with *p*_0_ kept at 102 and 112 mmHg, respectively. With respect to changes (∆) in augmentation index and backward pressure wave amplitude relative to the reference situation (i.e., the [*v*_s_*_,_*_LV_*↓−*REF], [*k↑−*REF], and [*v*_s_*_,_*_LV_*↓, k↑−*REF] differences), we found only a minor influence of increasing *p*_0_ (i.e., ∆AIx *≤* 8% and ∆${\hat{p}}_{\text{bw}}$ *≤* 5 mmHg, respectively).

We believe our model-based findings require further confirmation in clinical studies. Such a study could be a case-control design with isolated systolic hypertension and normotensive patient groups. Measurements in these studies should include speckle-tracking echocardiography to characterize LV contractile function (11), pulse wave analysis to characterize augmentation index (12), wave separation analysis (30) to characterize wave reflection behavior, as well as stroke volume and blood pressure measurements to estimate cardiac workload.

#### Conclusion.

We conclude that the AIx reflects both cardiac and vascular properties, and hence should not be considered a vascular parameter. Furthermore, an increase in AIx does not necessarily relate to increased stroke work. Hence, we believe AIx should be abandoned as a proxy for increased wave reflection magnitude due to an arterial stiffness increase.

## GRANTS

M.H.G. Heusinkveld was supported by a Kootstra Talent Fellowship from Maastricht University Medical Centre. A. Hughes and K. Reesink were supported by the British Heart Foundation (Grant PG29934). J. Lumens acknowledges support from the Dr. Dekker Program of the Dutch Heart Foundation (Grant 2015T082) and the Netherlands Organization for Scientific Research (NWO-ZonMw, VIDI Grant 016.176.340).

## DISCLOSURES

No conflicts of interest, financial or otherwise, are declared by the authors.

## AUTHOR CONTRIBUTIONS

M.H.H., T.D., A.D.H., and K.D.R. conceived and designed research; M.H.H. performed experiments; M.H.H., T.D., B.S., and K.D.R. analyzed data; M.H.H., T.D., B.S., and K.D.R. interpreted results of experiments; M.H.H. and B.S. prepared figures; M.H.H., W.H., and K.D.R. drafted manuscript; M.H.H., T.D., J.L., W.H., B.S., A.D.H., and K.D.R. edited and revised manuscript; M.H.H., T.D., J.L., W.H., B.S., A.D.H., and K.D.R. approved final version of manuscript.

## ENDNOTE

At the request of the authors, readers are herein alerted to the fact that additional materials related to this manuscript may be found at the GitHub of one of the authors, which at the time of publication they indicate is: https://github.com/Mheu1991/AugmentationIndex_SimulationStudy. These materials are not a part of this manuscript and have not undergone peer review by the American Physiological Society (APS). APS and the journal editors take no responsibility for these materials, for the website address, or for any links to or from it.
